# Luteolin Inhibits Human Prostate Tumor Growth by Suppressing Vascular Endothelial Growth Factor Receptor 2-Mediated Angiogenesis

**DOI:** 10.1371/journal.pone.0052279

**Published:** 2012-12-31

**Authors:** Poyil Pratheeshkumar, Young-Ok Son, Amit Budhraja, Xin Wang, Songze Ding, Lei Wang, Andrew Hitron, Jeong-Chae Lee, Donghern Kim, Sasidharan Padmaja Divya, Gang Chen, Zhuo Zhang, Jia Luo, Xianglin Shi

**Affiliations:** 1 Graduate Center for Toxicology, College of Medicine, University of Kentucky, Lexington, Kentucky, United States of America; 2 National Centre for Aquatic Animal Health, Cochin University of Science and Technology, Cochin, India; 3 Department of Internal Medicine, College of Medicine, University of Kentucky, Lexington, Kentucky, United States of America; Univ of Bradford, United Kingdom

## Abstract

Angiogenesis, the formation of new blood vessels from pre-existing vascular beds, is essential for tumor growth, invasion, and metastasis. Luteolin is a common dietary flavonoid found in fruits and vegetables. We studied the antiangiogenic activity of luteolin using *in vitro, ex vivo,* and *in vivo* models. *In vitro* studies using rat aortic ring assay showed that luteolin at non-toxic concentrations significantly inhibited microvessel sprouting and proliferation, migration, invasion and tube formation of endothelial cells, which are key events in the process of angiogenesis. Luteolin also inhibited *ex vivo* angiogenesis as revealed by chicken egg chorioallantoic membrane assay (CAM) and matrigel plug assay. Gelatin zymographic analysis demonstrated the inhibitory effect of luteolin on the activation of matrix metalloproteinases MMP-2 and MMP-9. Western blot analysis showed that luteolin suppressed VEGF induced phosphorylation of VEGF receptor 2 and their downstream protein kinases AKT, ERK, mTOR, P70S6K, MMP-2, and MMP-9 in HUVECs. Proinflammatory cytokines such as IL-1β, IL-6, IL-8, and TNF-α level were significantly reduced by the treatment of luteolin in PC-3 cells. Luteolin (10 mg/kg/d) significantly reduced the volume and the weight of solid tumors in prostate xenograft mouse model, indicating that luteolin inhibited tumorigenesis by targeting angiogenesis. CD31 and CD34 immunohistochemical staining further revealed that the microvessel density could be remarkably suppressed by luteolin. Moreover, luteolin reduced cell viability and induced apoptosis in prostate cancer cells, which were correlated with the downregulation of AKT, ERK, mTOR, P70S6K, MMP-2, and MMP-9 expressions. Taken together, our findings demonstrate that luteolin inhibits human prostate tumor growth by suppressing vascular endothelial growth factor receptor 2-mediated angiogenesis.

## Introduction

Angiogenesis, the process by which capillaries sprout from preexisting blood vessels, occurs in response to the increasing demand for nutrients and oxygen experienced by proliferating tumor cells. It plays a pivotal role in tumor growth, invasion and metastasis and is tightly regulated by a large number of proangiogenic and antiangiogenic factors. The angiogenic process involves the activation, proliferation, and migration of endothelial cells toward angiogenic stimuli produced by the tumor [1], [2]. Induced secretion of angiogenic factors is commonly observed in most aggressive tumors. Among various angiogenic factors, the most essential is vascular endothelial growth factor (VEGF), which exerts its mitogenic activity especially on endothelial cells [1].

VEGF exerts its biological effects by binding to its receptor tyrosine kinases, expressed on endothelial cells. The biologically relevant VEGF signaling events are mediated mainly via VEGF receptor 2 (VEGFR2) [3]. Activation of VEGFR2 leads to the activation of various downstream signal transduction proteins, including extracellular signal-related kinase (ERK) [4], [5], AKT [4], mammalian target of rapamycin (mTOR) [6], and ribosomal protein S6 kinase (p70S6K) [7], which promotes the growth, migration, differentiation and survival of endothelial cells in pre-existing vasculature.

Cytokines are low-molecular weight soluble proteins that transmit signals between cells and are involved in several disorders. Pro-inflammatory cytokines, including interleukin-1β (IL-1β), interleukin-6 (IL-6), interleukin-8 (IL-8), and tumor necrosis factor-alpha (TNF-α) are involved in these pathological processes [8]. Upregulation of these cytokines is closely linked to chronic inflammation and cancer [9]. These cytokines could be prometastatic or proangiogenic and their deregulated expression directly correlates with the metastatic potential of several human carcinomas [10]. There is a direct correlation between proinflammatory cytokines level and tumor angiogenesis. Increased serum levels of proinflammatory cytokines such as IL-1β, IL-6, and TNF-α were found in tumor angiogenesis induced animals [11]. Moreover altered levels of proinflammatory and proangiogenic factors are observed in various forms of cancer [12].

Degradation of the extracellular matrix and basement membrane is one of the first steps in angiogenesis. The matrix metalloproteinases (MMPs) belong to a family of endopeptidases that are capable of degrading basement membranes and components of the extracellular matrix. Increased expression of MMPs has been strongly implicated in tumor growth, invasion and metastasis [13]. Among diverse proteolytic enzymes, gelatinases such as MMP-2 and MMP-9 play a key role in degrading most ECM components surrounding tumor tissue [14] which are expressed in elevated levels in proliferating endothelial cells [14].

**Figure 1:**
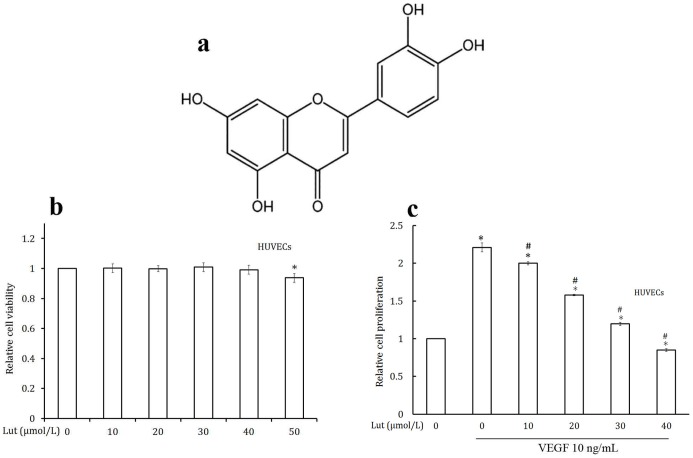
Luteolin inhibited the VEGF induced cell proliferation in HUVECs. (a) Chemical structure of luteolin. (b) Effect of luteolin on HUVECs viability in culture. Cell viability was quantified by MTT assay. Values are means ± SD (mean of triplicate). *p<0.05 denotes a statistically significant difference from untreated controls. (c) Luteolin inhibits the VEGF induced proliferation of endothelial cells. HUVECs were treated with different concentrations of luteolin and VEGF for 48 h. Relative cell proliferation was determined by MTT assay. Values are means ± SD (mean of triplicate). *p<0.05 denotes a statistically significant difference from untreated controls; #p<0.05 denotes a statistically significant difference from VEGF control.

Medicinal plants and phytochemicals are potential novel agents for developing antiangiogenic drugs. Many studies have reported the use of flavonoids as effective natural inhibitor on cancer initiation and progression [15]. Luteolin (3′,4′,5,7-tetrahydroxyflavone, Fig. 1A) is a common dietary flavonoid found in fruits, vegetables, and medicinal herbs. This compound possesses a variety of anti-tumor properties, such as the inhibition of proliferation and the induction of apoptosis [16], [17]. Luteolin (Lut) inhibited the migration and invasion of HGF-induced HepG2 cells by suppressing the phosphorylation of c-Met, ERK, and Akt in hepatoma [18] and it inhibited the invasion of cells in lung and breast cancers [19].

In the present study, we analyzed the effect of luteolin on the inhibition of tumor specific angiogenesis *in vitro, ex vivo,* and *in vivo* models. Our results show that luteolin could significantly inhibit VEGF-stimulated endothelial cell proliferation, chemotactic migration, invasion, tube formation, and tumor angiogenesis by targeting VEGFR-2-regulated AKT/ERK/mTOR/P70S6K/MMPs pathway, leading to the suppression of prostate tumor growth and tumor angiogenesis. Luteolin also suppressed angiogenesis as measured by chick embryo chorioallantoic membrane (CAM) assay *ex vivo* and rat aortic ring assay *in vitro.* Moreover, luteolin inhibited cancer growth by inducing apoptosis and inhibiting angiogenesis in human prostate xenograft mouse model.

## Materials and Methods

### Ethics Statement

Animals were handled in strict accordance with good animal practice as defined by Institutional Animal Care and Use Committee (IACUC), University of Kentucky and this study was carried out with their prior approval (Approval ID: 2011-0851).

### Chemicals and Reagents

Luteolin (>99% pure) was purchased from Sigma (St. Louis, MO, USA), dissolved in DMSO, aliquoted, and stored at −20°C. Bacteria-derived recombinant human VEGF (121 a.a.) was purchased from ProSpec-Tany TechnoGene Ltd. (Ness Ziona, Israel). Growth factor-reduced Matrigel was purchased from BD Biosciences (Bedford, MA). The antibodies anti-AKT, anti-mTOR, anti-p70S6K1, antipoly (ADP-ribose) polymerase (PARP), anti-cleaved caspase-3, phospho-specific anti-AKT (Ser473), anti-mTOR (Ser2448), anti-p70S6K1 (Thr421/Ser424), and anti-VEGFR2 (Tyr1175) were purchased from Cell Signaling Technology (Beverly, MA). The antibodies against phospho-specific ERK 1/2, ERK 1/2, MMP-2, MMP-9, and β-actin were obtained from Santa Cruz Biotechnology (Santa Cruz, CA).

### ELISA Kits

Highly specific quantitative ‘sand wich’ Elisa kits for human IL-1β, IL-6, IL-8, and TNF-α were purchased from BioLegend, Inc. (CA, USA) and the ELISA kit for VEGF was purchased from RayBiotech (GA, USA).

### Cell Lines and Cell Culture

HUVECs were purchased from American Type Culture Collection (ATCC, Manassas, VA) and grown in Clonetics Endothelial Cell Growth Medium-2 (EGM-2; Lonza, Walkersville, MD). Human prostate cancer (PC-3) cells were purchased from American Type Culture Collection and cultured in RPMI 1640 medium supplemented with 10% fetal bovine serum (FBS). HUVECs and PC-3 cells were cultured at 37°C under a humidified 95%: 5% (v/v) mixture of air and CO_2_.

### Cell Viability Assay

HUVECs or PC-3 cells were seeded (5000 cells/well) in 96-well flat bottomed titer plate and incubated for 24 h at 37°C in 5% CO_2_ atmosphere. Different dilutions of luteolin were added and incubated further for 48 h. Before 4 h completion of incubation, 10 µl MTT (5 mg/ml) was added [20]. The cultures were solubilized and spectrophotometric absorbance was measured at 595 nm using a microtiter plate reader. The number of viable cells was presented relative to untreated controls.

### Cell Proliferation Assay

HUVECs were seeded (5000 cells/well) in 96-well flat bottomed titer plate and incubated for 24 h at 37°C in 5% CO_2_ atmosphere. EGM-2 (0.5% FBS) containing 10 ng/mL VEGF with or without different dilutions of luteolin was added and incubated for 48 h. Relative cell proliferation was determined by MTT assay.

### Wound-healing Migration Assay

HUVECs were grown into wells of collagen coated 24 well plate dishes to 100% confluence. Cells were starved to inactivate cell proliferation and then wounded by pipette tips. EGM-2 containing 0.5% FBS was added with or without 10 ng/mL VEGF and different dilutions of luteolin. Images of the cells were taken after 24 h of incubation. Migrated cells were quantified manually, and presented relative to untreated controls. Three independent experiments were performed.

### Collagen Matrix Invasion Assay

Collagen matrix invasion assay was performed as described previously [2]. Briefly, HUVECs (10^5^ cells/Transwell) along with the indicated concentrations of luteolin were seeded into the upper compartment of invasion chambers. The bottom chambers were filled with 500 µL EGM-2 supplemented with 10 ng/mL VEGF. After 24 h incubation, migrated cells were fixed with 3.7% paraformaldehyde and stained with 0.5% crystal violet in 2% ethanol. Membranes were washed and the dye was eluted with 10% acetic acid. Absorbance was measured at 595 nm using a microtiter plate reader (Beckman coulter). The number of invaded cells was presented relative to untreated controls.

### Capillary-like Tube Formation Assay

HUVECs in medium EGM-2 were seeded into the matrigel layer in 24–well plates at a density of 6×10^4^ cells/well along with 10 ng/ml VEGF. Various dilutions of luteolin were added into the wells and incubated for 24 h at 37°C in 5% CO_2_ atmosphere. Tube formation was examined and photographed using an inverted microscope (20X) [21].

### ELISA Assays for VEGF and Pro-inflammatory Cytokines

PC-3 cells (2×10^5^) were plated in 24-well plates and allowed to attach by overnight incubation at 37°C. Cells were treated with desired concentrations of luteolin for 24 h. Subsequently, the culture media was collected and used to estimate the levels of VEGF and pro-inflammatory cytokines such as IL-1β, IL-6, IL-8, and TNF-α using commercially available kit according to the manufacturers’ recommendations.

### Rat Aortic Ring Assay

The rat aortic ring assay was used as the *in vitro* angiogenesis study model [11]. Dorsal aorta from a freshly sacrificed Sprague–Dawley rat was taken out in a sterile manner and rinsed in ice cold PBS. It was then cut into ∼1 mm long pieces using surgical blade. Each ring was placed in a collagen pre-coated 96-well plate. VEGF, with or without different dilutions of luteolin, was added to the wells. On day 6, the rings were analyzed by phase-contrast microscopy and microvessel outgrowths were quantified and photographed [22]. The assay was scored from 0 (least positive) to 5 (most positive) in a double-blind manner. Each data point was assayed 6 times [23].

### CAM Assay in Fertilized Chicken Eggs

The effect of luteolin on *ex vivo* angiogenesis was determined by CAM assay. Briefly, fertile leghorn chicken eggs (Poultry Breeding farm, University of Kentucky) were candled on embryonic day 8; a small opening was made at the top of the live eggs. Luteolin for treatment was mixed with 0.5% methyl cellulose in water and gently placed on the CAM. The eggs were incubated for 48 h and photographed. Blood vessels density was quantified by Image J software and represented as a bar diagram.

### Matrigel Plug Assay

Fertilized chicken eggs (Poultry Breeding farm, University of Kentucky) were incubated at 37°C for 9 days, and angiogenesis assay was performed as previously described [24]. In brief, VEGF (100 ng) and luteolin (10 µg) were mixed with matrigel and implanted into the CAM at day 9. After 96 h of incubation, the tumor plugs were taken out and dispersed in PBS and incubated at 4°C overnight. Hemoglobin levels were determined using Drabkin’s reagent (Sigma-Aldrich, St. Louis, MO) according to manufacturer instructions.

### Gelatin Zymography

SDS-PAGE was performed with 0.1% gelatin incorporated in the separating gel [25]. HUVECs of subconfluent cultures were incubated with serum free medium for 24 h at 37°C in 5% CO2 atmosphere. The conditioned medium was then collected and subjected to zymographic analysis. Fifty microliters of sample (equivalent to 100 µg protein) was activated with 5 µl trypsin solution (75 µg/ml) in the presence and absence of luteolin (10, 20, and 40 µM) in 100 mM Tris–HCl, 10 mM CaCl_2_ buffer (pH- 8.0) and incubated for 1 h at room temperature. Samples were mixed with an equal volume of 2X sample buffer and separated using 12% polyacrylamide gels containing 0.1% gelatin. The zymograms were incubated in 100 mM Tris (pH-8.0), 10 mM CaCl_2_ for 12 h at 37°C followed by staining with Gelcode Blue stain reagent for 2 h. Gels were destained to visualize the clear area against the dark background.

### Assessment of Apoptosis

The extent of apoptosis in prostate cancer cells was evaluated by flow cytometric analysis using FITC conjugated Annexin V/propidium iodide (BD PharMingen, San Diego, CA) staining according to the manufacturer’s instructions. Both early apoptotic (Annexin V-positive, PI-negative) and late apoptotic (Annexin V-positive and PI-positive) cells were included in cell death determinations.

### Western Blot Analysis

To determine the effects of luteolin on the VEGFR2–dependent mTOR signaling pathway, HUVECs were first starved in serum-fee EGM-2 for 6 h, and then pretreated with or without luteolin for 1 h, followed by the stimulation with 50 ng/mL of VEGF for 10 min (for VEGFR2 activation) or 30 min (for mTOR pathway kinase activation). To examine mTOR pathway in prostate tumor cells, normal cultured PC-3 cells were directly treated with indicated dilutions of luteolin for 6 h. The total cellular samples were washed twice with ice-cold PBS and lysed in 1X NuPAGE LDS sample buffer supplemented with 50 mM dithiothreitol. The protein concentration was determined using Coomassie Protein Assay Reagent (Pierce, Rockford, IL). About 40 µg cellular proteins were separated using 6%–12% SDS-polyacrylamide gel and transferred to nitrocellulose membrane. Membranes were blocked with 5% fat-free dry milk in 1X Tris-buffered saline (TBS) and incubated with antibodies. Protein bands were detected by incubating with horseradish peroxidase-conjugated antibodies (Kirkegaard and Perry Laboratories, Gaithersburg, MD) and visualized with enhanced chemiluminescence reagent (Perkin Elmer, Boston, MA).

For tissue sections, radioimmunoprecipitation assay (RIPA) buffer was added to the sections and homogenized with electric homogenizer. After incubation for 20 minutes on ice, samples were centrifuged for 30 minutes at 12,000 rpm at 4°C and supernatant was collected as total cell lysate. SDS-PAGE was carried out as described previously [26].

### Xenograft Human Prostate Tumor Mouse Model

Six week old male BALB/cA nude mice were purchased from Charles River Laboratories (Wilmington, MA). Animals were housed in a specific pathogen-free room within the animal facilities at the University of Kentucky, Lexington, KY. All animals were allowed to acclimatize to their new environment for one week prior to use and were handled according to the Institutional Animal Care and Use, University of Kentucky. Mice were randomly divided into 2 groups (5 animals/group). PC-3 cells (5×10^6^ cells per mouse) were resuspended in serum-free RPMI-1640 medium with matrigel basement membrane matrix (BD Biosciences) at a 1∶1 ratio (total volume: 100 µL) and then were subcutaneously injected into the flanks of nude mice. After tumors grew to about 100 mm^3^, mice were treated intraperitoneally with or without luteolin (10 mg/kg/d). The body weight of each mouse was recorded and tumor volume was determined by Vernier caliper every day, following the formula of *A×B^2^×0.52*, where A is the longest diameter of tumor and B is the shortest diameter. After 16 d, the mice were killed and solid tumors were removed.

### Histology and Immunohistochemistry

Tumor tissues were fixed in 10% neutral-buffered formalin for 24 hours, processed, and embedded in paraffin blocks. The sections (5 µm) were blocked with 10% goat serum and incubated with a rabbit anti-CD31 (1∶100; Novus Biologicals Inc, Littleton, CO) and mouse ant-CD34 (1∶100; BD Pharmingen Inc, San Diego, CA) antibodies for 24 h. The slides were subsequently incubated for 30 min with biotinylated anti-rabbit/anti-mouse secondary antibody (Vector laboratories, Burlingame, CA) and followed by incubation of Vectastain ABC Kit (Vector Laboratories). Diaminobenzidine (Sigma) was used as the chromagen and methyl green (Sigma) as the counterstain.

### Statistics

The values were presented as means ± SD. Two-way analysis of variance (ANOVA) and Student’s t test were used for statistical analysis. p<0.05 was considered significantly different.

## Results

### Effect of Luteolin on HUVECs Viability in Culture

Cell viabilty was determined by MTT assay. Effect of luteolin on HUVECs viability in culture is shown in Figure 1B. luteolin was found to be non-toxic to HUVECs at concentrations of 10–40 µmol/L and these concentrations were used for further *in vitro* experiments.

### Luteolin Inhibited HUVECs Proliferation, Chemotactic Migration, Invasion, and Tube Formation

HUVECs showed very high rate of proliferation when stimulated with VEGF. Treatment with luteolin (10–40 µmol/L) significantly inhibited VEGF induced proliferation of HUVECs (Fig. 1C).

**Figure 2:**
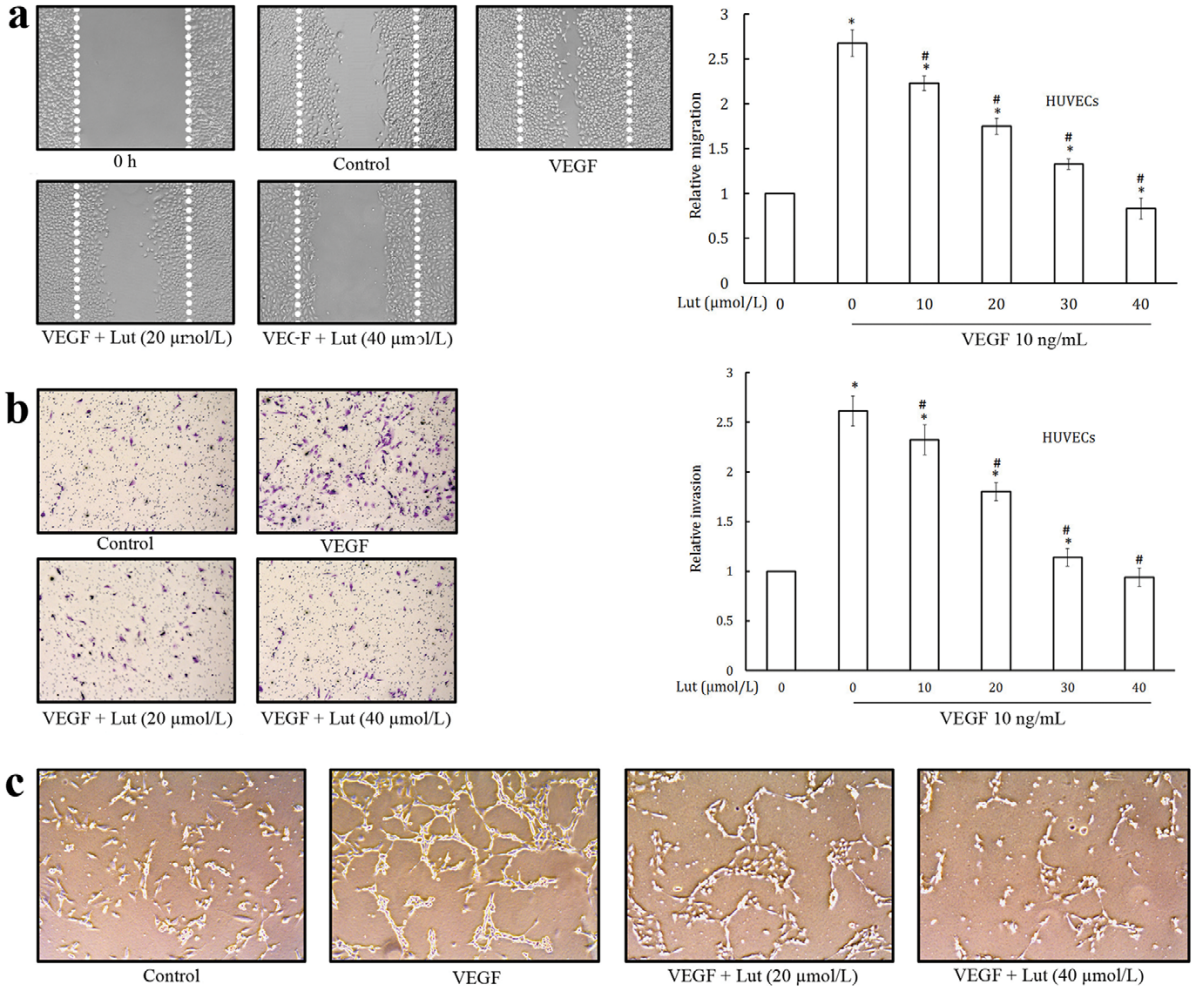
Luteolin inhibited VEGF-induced migration, invasion, and tube formation of endothelial cells. (a) Luteolin inhibited HUVECs migration. Cells were starved to inactivate cell proliferation and then wounded by pipette tips. EGM-2 with or without 10 ng/mL VEGF and different dilutions of luteolin were added. Migrated cells were quantified by manual counting. (b) Luteolin inhibited HUVECs invasion. HUVECs with the indicated concentrations of luteolin were seeded into the upper compartment of invasion chambers. The bottom chambers were filled with EGM-2 supplemented with VEGF. After 24 h incubation, migrated cells were fixed, stained and quantified. (c) Luteolin inhibited the tube formation of HUVECs. HUVECs were treated with various dilutions of luteolin with VEGF for 24 h, cells were fixed, and tubular structures were photographed. Values are means ± SD (mean of triplicate). *p<0.05 denotes a statistically significant difference from untreated controls; #p<0.05 denotes a statistically significant difference from VEGF control.

Cell migration is essential for angiogenesis in endothelial cell [27]. Effect of luteolin on the chemotactic motility of HUVECs is shown in Figure 2A. HUVECs migrated into the clear area when stimulated with VEGF. Luteolin significantly inhibited the VEGF induced migration of endothelial cells in a dose dependent manner. Almost complete inhibition of endothelial cell migration was observed at 40 µmol/L of luteolin which was comparable to that at 0 h incubation. This concentration is non-toxic as is evident from MTT assay (Fig. 1B) and hence the inhibitory effect could not be attributed to cytotoxicity.

HUVECs showed a high invasive property through the collagen matrix when stimulated with VEGF (Fig. 2B). Large numbers of cells were found on the lower surface of the polycarbonate, but treatment with luteolin produced a significant inhibition in the invasion of the collagen matrix by HUVECs in a dose dependent manner.

Tube formation of endothelial cells is one of the key steps of angiogenesis [27]. Treatment of HUVECs with luteolin significantly inhibited tube formation (Fig. 2C). Incubation of HUVECs on matrigel with VEGF resulted in the formation of elongated and tube like structures which were effectively reduced by luteolin (20 and 40 µmol/L).

### Luteolin Inhibited *ex vivo* Angiogenesis in CAM Assay

**Figure 3:**
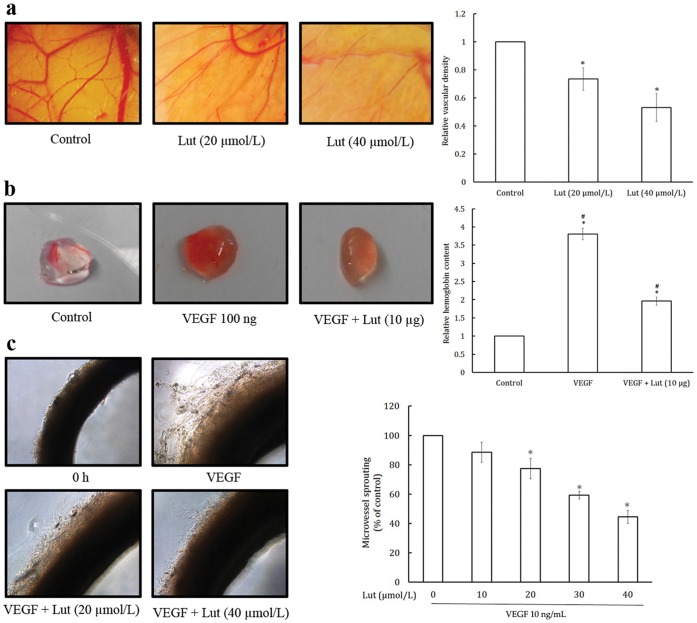
Luteolin inhibited *ex vivo* angiogenesis by CAM and matrigel plug assay and *in vitro* angiogenesis by rat aortic ring assay. (a) Luteolin inhibits *ex vivo* angiogenesis in CAM assay. Fertile leghorn chicken eggs were candled on embryonic day 8; a small opening was made at the top of the live eggs. Luteolin for treatment was mixed with 0.5% methyl cellulose in water and gently placed on the CAM. The eggs were incubated for 48 h and photographed. Blood vessels density was quantified by Image J software and represented as a bar diagram. Values are means ± SD (mean of triplicate). *p<0.05 denotes a statistically significant difference from untreated controls; #p<0.05 denotes a statistically significant difference from 20 and 40 µmole/L luteolin. (b) Luteolin inhibits *ex vivo* angiogenesis in matrigel plug assay. Matrigel plug containg VEGF and luteolin were implanted into the CAM at day 9 of fertilized chicken eggs. After 96 h of incubation, the matrigel plugs were taken out and dispersed in PBS and incubated at 4°C overnight. Hemoglobin levels were determined using Drabkin’s reagent according to manufacturer instructions. Values are means ± SD (mean of triplicate). *p<0.05 denotes a statistically significant difference from untreated controls; #p<0.05 denotes a statistically significant difference from VEGF control. (c) Luteolin inhibits microvessel outgrowth from the rat aortic ring. Dorsal aorta from a freshly sacrificed Sprague–Dawley rat was cut into ∼1 mm long pieces using surgical blade. Each ring was placed in a collagen pre-coated 96-well plate. VEGF, with or without different dilutions of luteolin, was added to the wells. On day 6, the rings were analyzed by phase-contrast microscopy and microvessel outgrowths were quantified and photographed. Values are means ± SD (mean of triplicate). *p<0.05 denotes a statistically significant difference from untreated controls.

CAM assay was used to determine the antiangiogenic effect of luteolin *ex vivo*. CAM revealed highly vascularized structure in the control group (Fig. 3A). Exposure to luteolin (20 and 40 µmol/egg) drastically reduced the vascular density which confirmed the anti-angiogenic potential of luteolin through *ex vivo* assay.

### Luteolin Inhibited *ex vivo* Angiogenesis in Matrigel Plug Assay

To confirm the anti-angiogenesis effects of luteolin *ex vivo*, matrigel plug assay was performed. As shown in Figure 3B, luetolin (10 µg) significantly inhibited VEGF-induced angiogenesis in the matrigel plug, indicating that luteolin effectively inhibited angiogenesis *ex vivo*. Hemoglobin level was also significantly lower in the luteolin treated matrigel plug, further confirming its antiangiogeneic potential.

### Luteolin Inhibited Microvessel Outgrowth from the Rat Aortic Ring

The inhibitory effect of luteolin on angiogenesis *in vitro* was assessed by aortic ring assay. VEGF induced microvessel outgrowth in rat aorta ring. As shown in Figure 3C, luteolin at 20 and 40 µmol/L inhibited micro-vessel growth after 6 days of incubation, indicating that luteolin inhibits angiogenesis *in vitro*.

### Luteolin Inhibited the Activation of VEGFR2-mediated Signaling Pathways

**Figure 4:**
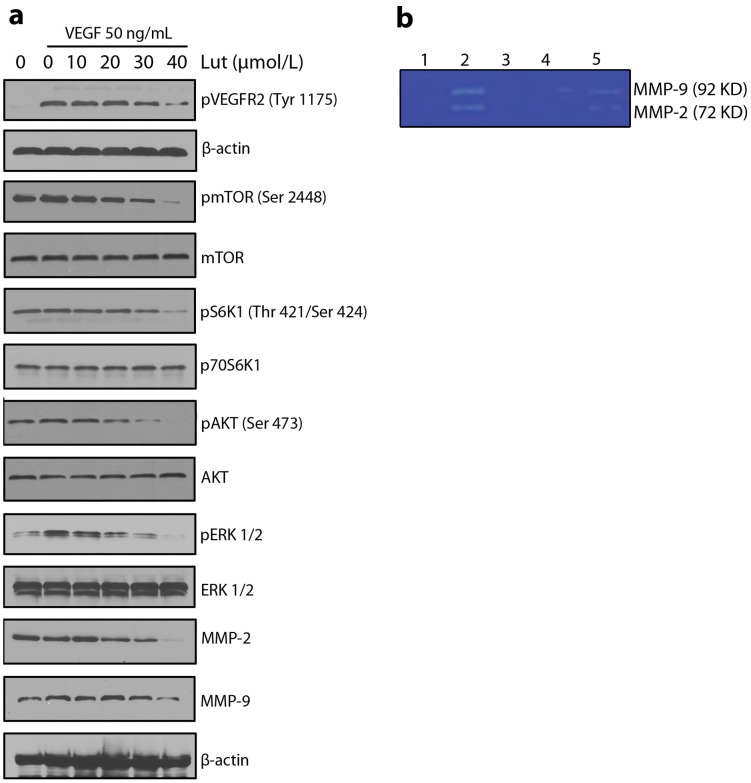
Luteolin inhibited the activation of VEGFR2-mediated signaling pathways. (a) Luteolin suppressed the activation of VEGFR2 and their downstream signaling pathway triggered by VEGF in HUVECs. Proteins from different treatments was tested by Western blotting and probed with specific antibodies. Experiments were repeated for three times. (b) Luteolin inhibits the MMP-2 and MMP-9 production by HUVECs. 1) Condition medium from untreated HUVECs without trypsin activation. 2) Condition medium from untreated HUVECs after trypsin activation. 3) Condition medium from untreated HUVECs after trypsin activation+EDTA. 4) Condition medium from pretreated HUVECs (40 µmol/L luteolin) after trypsin activation. 5) Condition medium from pretreated HUVECs (20 µmol/L luteolin) after trypsin activation.

Interaction of VEGFR2 with VEGF led to the activation of various downstream signaling molecules responsible for endothelial cell migration, proliferation, and survival [28]. To understand the molecular mechanism of luteolin-mediated anti-angiogenic properties, some key kinases involved in VEGFR2-mediated signaling pathways were assayed using Western blotting. Luteolin reduced phosphorylation of VEGFR2 in a dose-dependent manner (Fig. 4A). Luteolin at 40 µM significantly inhibited the activation AKT, ERK, mTOR, p70S6K, MMP-2, and MMP-9 (Fig. 4A), suggesting that luteolin exerted its anti-angiogenic activity through regulating the activation of VEGFR2-mediated downstream signaling cascade in endothelial cells. The concentrations of luteolin used for the above experiments were found to be non-toxic to endothelial cells (Fig. 1B), suggesting that the effect is not through decreasing the cell viability.

### Gelatin Zymography

The major forms of proteases detected by gelatin zymography are type IV collagenases, MMP-2 and MMP-9 (Fig. 4B). Since these proteases are secreted into the conditioned medium as proenzymes (latent form requiring activation), an activation step (trypsin activation) is necessary to get the MMPs activated. Conditioned medium after trypsin activation showed digested clear areas at 92 kDa and 72 kDa which were identical to MMP-9 and MMP-2 activity (Fig. 4B.2). Gels loaded with conditioned medium without trypsin activation, did not show clear degradative areas, indicating the inactive form of the enzyme (Fig. 4B.1). Trypsin activated conditioned medium loaded gels, when incubated with 10 mM EDTA (EDTA inhibits the proteolytic activity by chelating Ca+) did not show clear degradative areas, indicating that the enzyme responsible for degradation is metalloproteinase (Fig. 4B.3). Conditioned medium treated with luteolin at concentration of 40 µmol/L during trypsin activation did not show any clear bands (Fig. 4B.4) suggesting that luteolin inhibited the activation of procollagenase to active collagenase of metalloproteinases. But luteolin at 20 µmol/L showed diminished activation of metalloproteinases (Fig. 4B.5).

### Luteolin Inhibited Cell Viability, Migration, and Invasion of Prostate Cancer Cells

**Figure 5:**
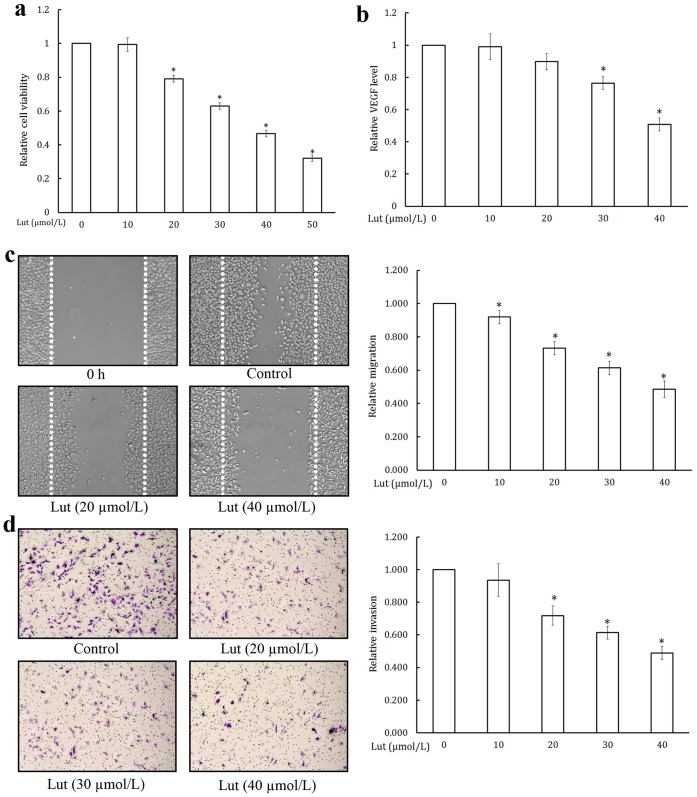
Luteolin inhibited cell viability, migration, and invasion of prostate cancer cells. (a) Luteolin inhibited cell viability of PC-3 cells. Cell viability was quantified by MTT assay. Values are means ± SD (mean of triplicate). *p<0.05 denotes a statistically significant difference from untreated controls. (b) Luteolin inhibited VEGF secretion in PC-3 cells. VEGF level was estimated by ELISA method. Values are means ± SD (mean of triplicate). *p<0.05 denotes a statistically significant difference from untreated controls. (c) Luteolin inhibited PC-3 cells migration. Cells were starved to inactivate cell proliferation and then wounded by pipette tips. Various dilutions of luteolin were added into the wells and incubated for 24 h; migrated cells were quantified by manual counting. (d) Luteolin inhibited HUVECs invasion. HUVECs with the indicated concentrations of luteolin were seeded into the upper compartment of invasion chambers. The bottom chambers were filled with serum-free RPMI. After 24 h incubation, migrated cells were fixed, stained and quantified.

Effect of luteolin on cell viability toward PC-3 cells in culture is shown in Figure 5A. At concentrations of 20–50 µmol/L luteolin significantly reduced the cell viability of PC-3 cells in a dose dependent manner.

Inhibition of tumor cell migration by luteolin is shown in Figure 5C. Luteolin significantly inhibited the migration of PC-3 cells towards the scraped clear area in a dose-dependent manner.

Prostate cancer cells showed a very high invasive property through the collagen matrix. Large numbers of cells were found in the lower surface of the polycarbonate membrane, but treatment with luteolin produced a significant inhibition in the invasion of the collagen matrix by the tumor cells in a dose-dependent manner (Fig. 5D).

### Luteolin Inhibited VEGF and Pro-inflammatory Cytokine Production in PC-3 Cells

**Figure 6:**
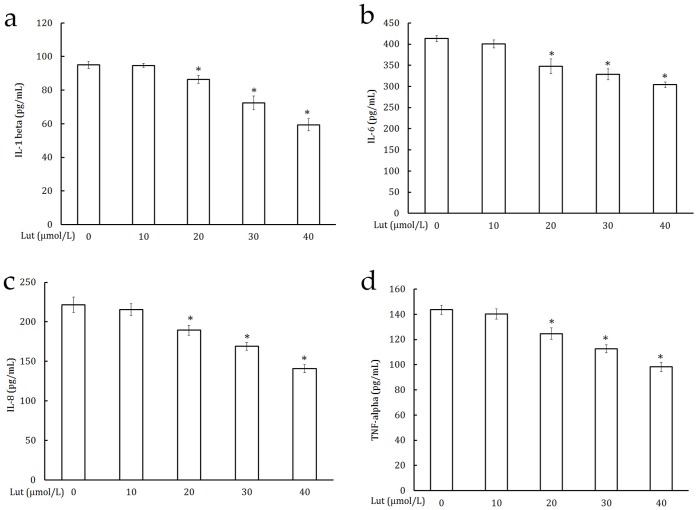
Luteolin inhibited pro-inflammatory cytokine production in PC-3 cells. Pro-inflammatory cytokines such as IL-1β, IL-6, IL-8, and TNF-α level were estimated by ELISA method according to the manufacturers’ recommendations. (a) IL-1β, (b) IL-6, (c) IL-8, and (c) TNF-α. Values are means ± SD (mean of triplicate). *p<0.05 denotes a statistically significant difference from untreated controls.

VEGF plays an important role in angiogenesis by promoting endothelial cell proliferation, migration, and differentiation [21]. The effect of luteolin on VEGF secretion and the results are shown in Figure 5B. The luteolin treatment caused a dose-dependent and statistically significant decrease in VEGF secretion. Effect of luteolin on proinflammatory cytokines production is shown in Figure 6. Luteolin significantly inhibited the production of IL-1β (Fig. 6A), IL-6 (Fig. 6B), IL-8 (Fig. 6C), and TNF-α (Fig. 6D) by PC-3 cells in culture.

### Luteolin Potentiated Apoptosis in Prostate Cancer Cells

**Figure 7:**
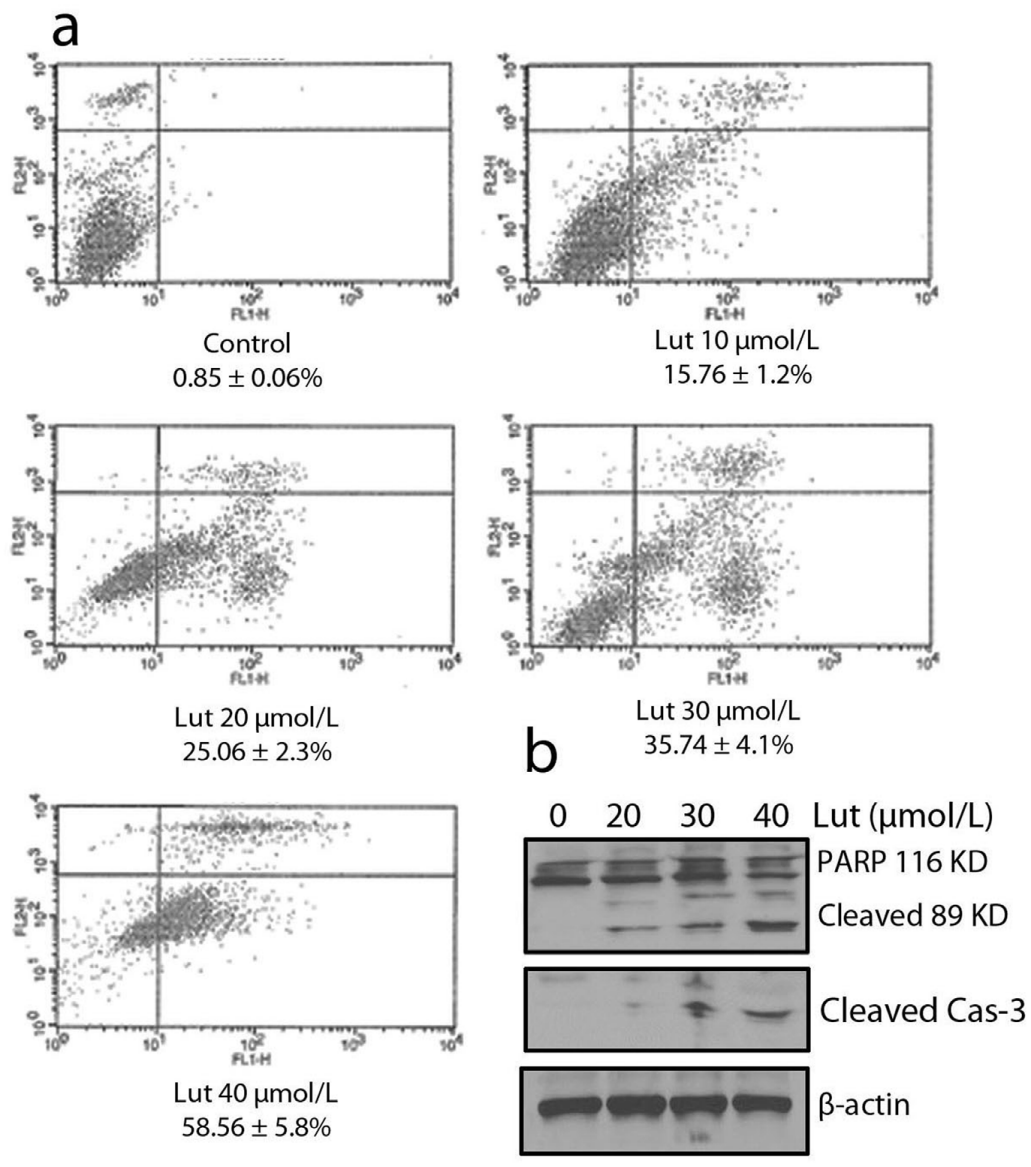
Luteolin induced apoptosis in prostate cancer cells. (a) Cells were stained with Annexin V/PI, and apoptosis was determined using flow cytometry. PC-3 cells were treated with various concentrations of luteolin for 48 h, after which apoptosis was determined by annexin V/PI staining with flow cytometry. (b) Luteolin induced PC-3 cancer cell apoptosis by the cleaved-PARP and Caspase-3 analysis. PC-3 cells were treated with luteolin for 48 h, and whole cell proteins were analysed by Western blotting with antipoly (ADP-ribose) polymerase (PARP) and anti-cleaved caspase-3.

To determine whether the reduction of prostate cancer cell viability is related to apoptosis, FACS analysis was carried out. PC-3 cells were treated with various concentrations of luteolin (10–40 µmol/L) for 24 h and subjected to flow cytometry analysis after staining with annexin V-FITC and propidium iodide (PI). As shown in Figure 7A, the percentages of apoptotic cells increased in a dose-dependent manner.

To further verify the FACS analysis data, Western blotting analysis was performed to examine caspase-3 and PARP cleavage in cells treated with various concentrations of luteolin (20, 30, 40 µmol/L). Poly (ADP-ribose) polymerase (PARP) is a family of proteins involved in cell death [29], which is cleaved into 89- and 24-kD fragments during drug induced apoptosis [28]. Caspase-3, a member of the caspase family of 13 aspartate-specific cysteine proteases that play a central role in the execution of the apoptotic program [30], [31], is primarily responsible for the cleavage of PARP during cell death [32]. We found that treatment with luteolin (20, 30, 40 µmol/L) led to the activation of caspase-3 and subsequently to the cleavage of PARP from its intact form (116 kD) to its cleaved form (89 kD) markedly in PC-3 cells (Fig. 7B), Taken together, these results suggest that luteolin induces apoptosis in prostate cancer cells through a caspase-3-dependent pathway.

### Luteolin Inhibited AKT/ERK/mTOR/P70S6K/MMPs Pathway in PC-3 Prostate Cancer Cells

**Figure 8:**
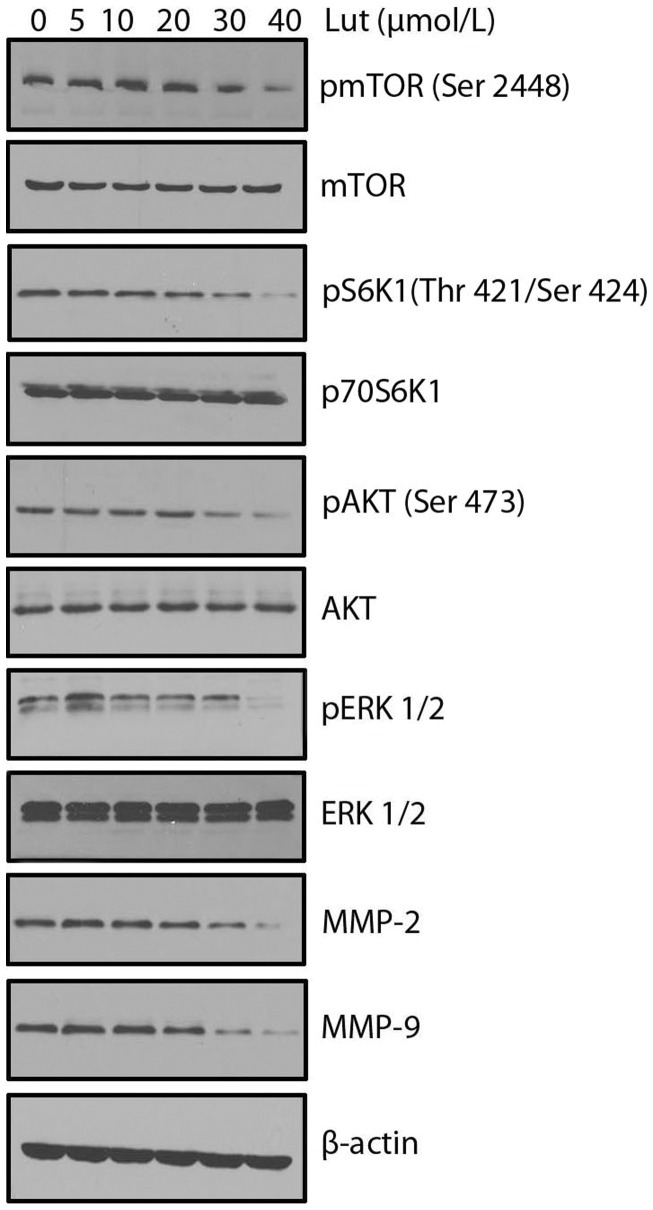
Luteolin inhibited the activation of AKT/ERK/mTOR/p70S6K/MMP-2/MMP-9 pathway in prostate cancer cells. Luteolin inhibited the activation of AKT/ERK/mTOR/p70S6K/MMP-2/MMP-9 pathway in PC-3 cells. Proteins from different treatments was tested by Western blotting and probed with specific antibodies. Experiments were repeated for three times.

AKT/mTOR/p70S6K signaling has been identified as a novel, functional mediator in angiogenesis [33], [34]. ERK1/2, one of the major targets of the mitogen-activated protein kinase (MAPK) signaling pathway, has been implicated in the regulation of angiogenesis for various functions including cell proliferation, migration and survival [35]. MMPs-2 and 9 are over expressed in many metastatic cancers and helps in the invasion of both tumor and endothelial cells [36]. In this study, it was found that luteolin supressed the activation of AKT, ERK, mTOR, P70S6K, MMP-2, and MMP-9 in a concentration-dependent manner in PC-3 cancer cells (Fig. 8).

### Luteolin Inhibited Tumor Angiogenesis and Tumor Growth *in vivo*

**Figure 9:**
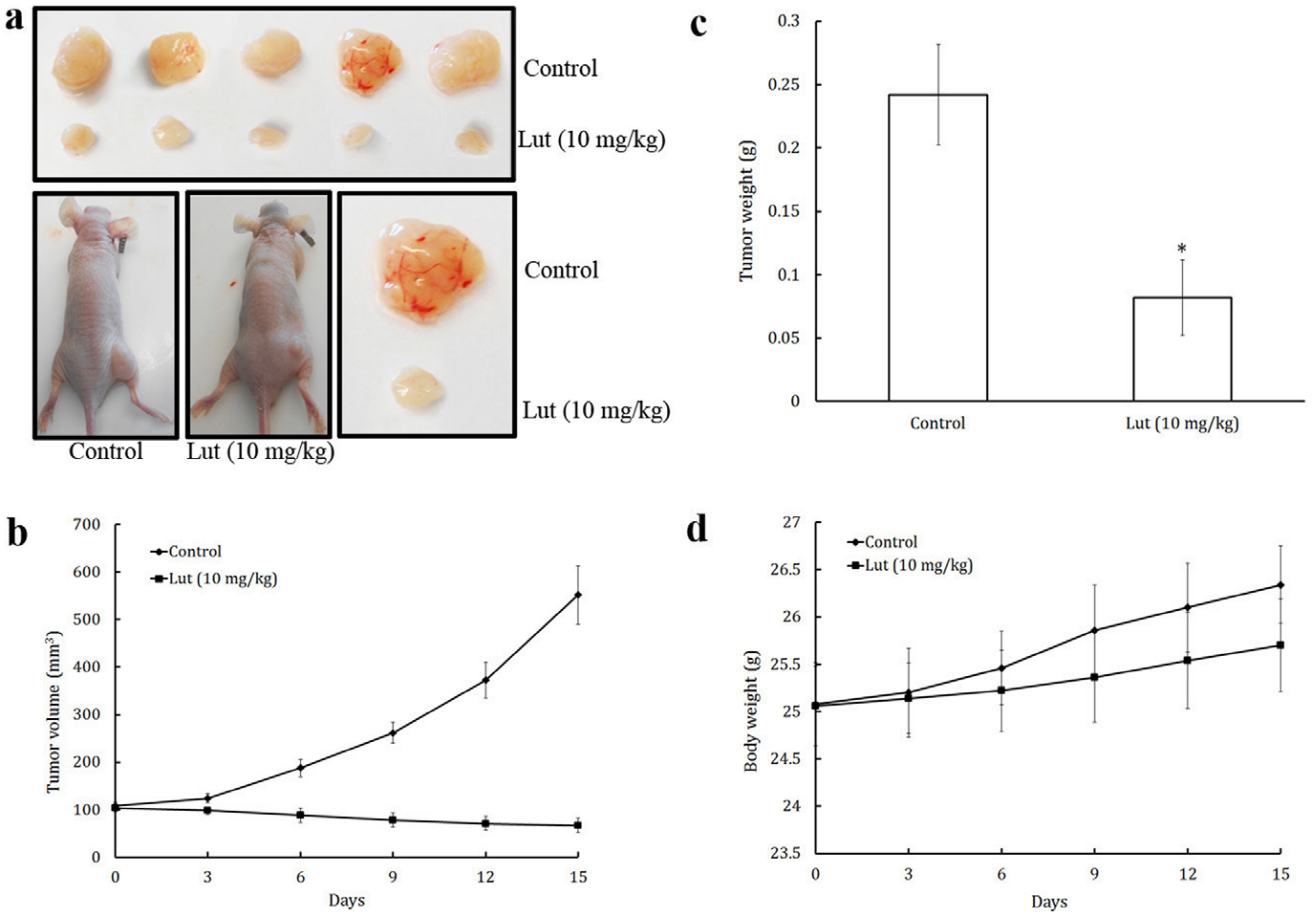
Luteolin inhibited tumor growth in a xenograft mouse model. PC-3 cells were injected into 6-week old BALB/cA nude mice (5×10^6^ cells per mouse). After tumors grew to about 100 mm^3^, mice were treated intraperitoneally with or without luteolin (10 mg/kg/d). (a) Solid tumors in the luteolin treated mice were significantly smaller than those in the control mice. Luteolin significantly reduced (b) tumor volume, and (c) tumor weight, (d) but had no effect on the body weight of mice. Values are means ± SD (mean of triplicate). *p<0.05 denotes a statistically significant difference from untreated controls.

Angiogenesis, the key step in tumor growth and metastasis, provides necessary oxygen and nutrients for the tumor [37]. A xenograft prostate tumor model was used to investigate the effect of luteolin on tumor growth and angiogenesis. PC-3 prostate cancer cells were injected (5×10^6^ per mouse) into the 6-week-old male BALB/cA nude mice. After the tumors had developed (about 100 mm^3^), the mice were injected with or without 10 mg/Kg/day luteolin (ip) every day (Fig. 9A). This intraperitoneal administration of luteolin significantly decreased tumor volume (Fig. 9B) and tumor weight (Fig. 9C) but had no effect on the body weight of mice (Fig. 9D). As shown in Figure 8B, tumors in control group increased from 108.31±7.35 to 551.66±61.32 mm^3^, whereas tumors in luteolin-treated group decreased from 102.97±25.05 to 66.84±4.72 mm^3^. The average weight of tumors from the control group was 0.242±0.04 gram whereas the average weight in luteolin treated group was only 0.082±0.03 gram, suggesting strong anti-tumor potential of luteolin in xenograft mouse prostate tumor model.

**Figure 10:**
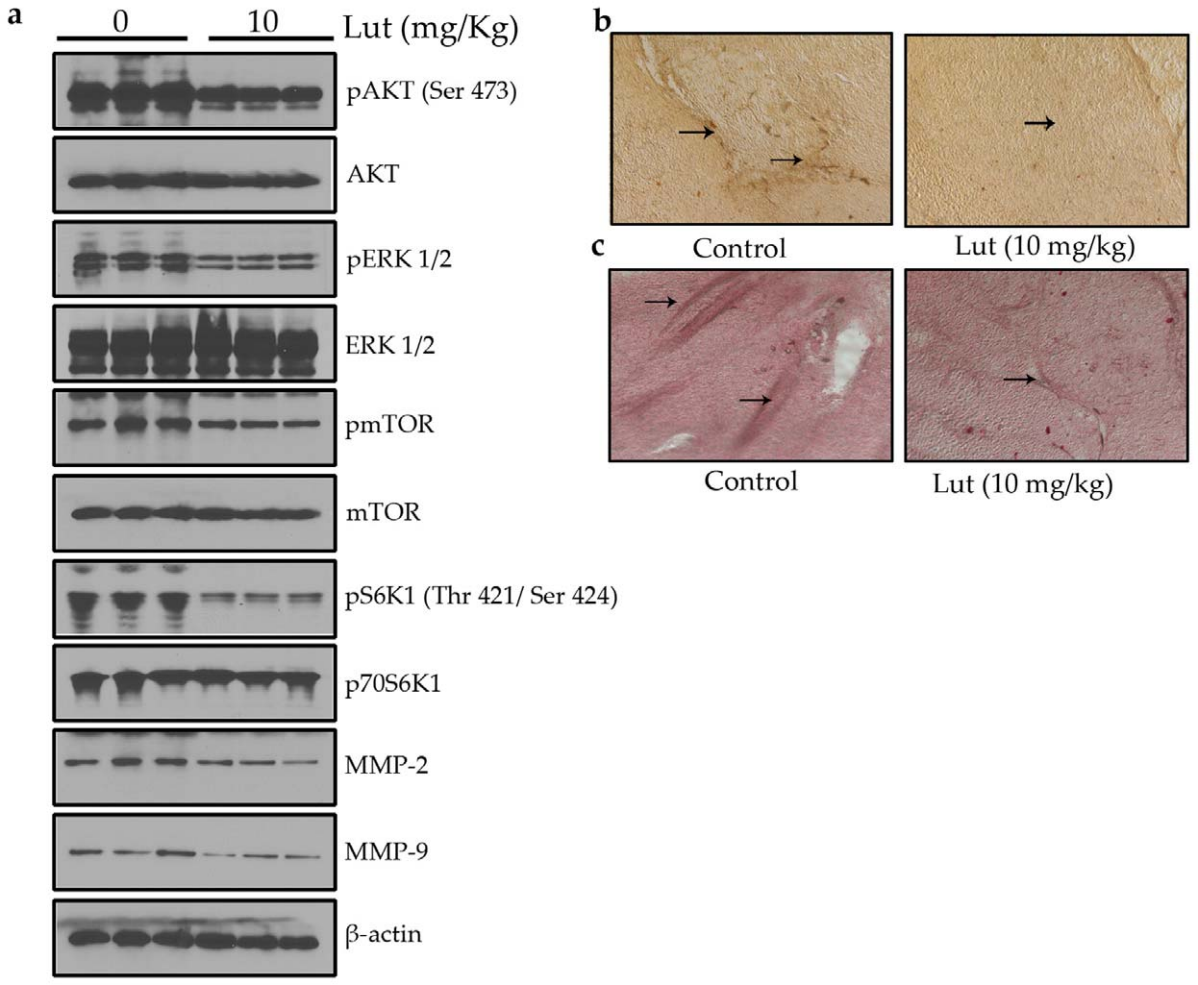
Luteolin inhibited tumor growth and tumor angiogenesis *in vivo* by suppressing AKT/ERK/mTOR/p70S6K/MMP-2/MMP-9 pathway. (a) Luteolin inhibited the activation of AKT/ERK/mTOR/p70S6K/MMP-2/MMP-9 pathway *in vivo*. Proteins from tumor tissue was tested by Western blotting and probed with specific antibodies. Experiments were repeated for three times. Luteolin inhibited tumor angiogenesis as evident from (b) CD31 and (c) CD34 immunohistochemistry. Tumor sections (5 µm) were incubated with a rabbit anti-CD31 and mouse anti-CD34 antibodies and were subsequently incubated with biotinylated anti-rabbit/anti-mouse secondary antibody, followed by staining with Vectastain ABC Kit.

To further investigate whether luteolin inhibited tumor growth by suppressing tumor angiogenesis, Western blot and immunohistochemical analysis of solid tumors were performed (Fig. 10). Tumors from luteolin treated animals showed suppressed activation of AKT, ERK, mTOR, P70S6K, MMP-2, and MMP-9 proteins (Fig. 10A). A large number of CD31 (Fig. 10B) and CD34 (Fig. 10C) positive cells were observed in untreated control group whereas a small number in luteolin treated group. All these observations indicate the antiangiogenic efficacy of luteolin.

## Discussion

Tumors can grow up to ∼2 mm size without requirement of blood supply as diffusion is sufficient at this level to support the removal of wastes from and supply of nutrients to tumor cells. Therefore, the angiogenesis process is an important target to suppress tumor growth and metastasis [38]. Angiogenic sprouting of blood vessels is completed in three steps, namely, proliferation of endothelial cells, breakdown of the extracellular matrix, and migration of endothelial cells. The induction of this process is mediated by angiogenic factors released by both tumor and host cells, and depends on the net balance of positive and negative regulators. These angiogenic factors bind to receptors on nearby blood vessels and induce the activation, proliferation, and migration of endothelial cells toward the tumor [39]. Accordingly, inhibition of angiogenesis is an attractive approach to treat cancer [40].

The degradation of the vascular basement membrane, which is required for endothelial cell invasion, is performed by type IV collagenases, called matrix metalloproteinases (MMPs) [41]. MMPs are a broad family of zinc-binding endopeptidases that play a key role in the extracellular matrix (ECM) degradation associated with cancer cell invasion, metastasis, and angiogenesis [42]. MMPs degrade collagen and other extracellular matrix components, disrupting the basement membrane barrier, and enabling endothelial cells to migrate from preexisting vessels toward angiogenic stimuli and to proliferate [43]. In particular, MMP-2 and MMP-9 play an important role in the angiogenic response as demonstrated in ECs (endothelial cells) as well as *in vivo* animal models [44]. Overexpressions of MMP-2 and MMP-9 have been demonstrated in human colorectal cancers [45]. Several experiments have confirmed the key role of these enzymes in angiogenesis [46], [47]. These enzymes are secreted in inactive proenzymatic forms, which require activation. It has been shown that trypsin can activate pro-MMPs to active MMPs [48].

Luteolin significantly inhibited the endothelial cell proliferation, invasion, migration, and tube formation. MMP-2 and MMP-9 expression and activation were downregulated by the treatment of luteolin, similar results were observed by the treatment with curcumin, another important naturally occurring compound [49]. In addition, luteolin treatment significantly inhibited neovascularization *ex vivo* in CAM assay and matrigel plug assay. Luteolin also exhibited cytotoxicity and induced apoptosis towards prostate tumor cells (PC-3). In the present study we have found that intraperitoneal administration of luteolin significantly decreased volume and weight of tumors, but had no effect on the body weight of mice. Immunohistochemical data was also showed that the expressions of endothelial cell markers, CD31 and CD34 were markedly less in tumor sections of luteolin treated animals.

Several inflammatory cytokines have been linked with tumorigenesis, which suggests that inflammation is associated with cancer development [50]. Tumor necrosis factor (TNF)-α is a potent pleiotropic proinflammatory cytokine produced by macrophages, neutrophils, fibroblasts, keratinocytes, NK cells, T and B cells, and tumor cells [51]. The role of TNF-α has been linked to all steps of tumorigenesis, including cellular transformation, promotion, survival, proliferation, invasion, angiogenesis, and metastasis [9]. Serum TNF-α level was found to be elevated in cancer patients [52]. IL-1β is a proinflammatory pleiotropic cytokine that has been shown to be an endogenous mediator of various acute and chronic inflammatory conditions. Furthermore IL-1β promotes the production of angiogenic proteins from host stromal or infiltrating cells in tumor microenvironment enhancing tumor growth and metastasis [53], [54]. Interleukin-6 (IL-6), a multifunctional cytokine, induces angiogenesis *ex vivo* and *in vivo*. In addition, *in vitro* studies have shown that IL-6 is capable to increase endothelial permeability and stimulates the proliferation of endothelial cells [55]–[57]. Interleukin-8 (IL-8) has been shown to play an important role in tumor growth, angiogenesis, and metastasis [58], [59]. Luteolin treatment significantly decreased the elevated levels of proinflammatory cytokines, IL-1β, IL-6, IL-8, and TNF-α in PC-3 cells.

Activation of endothelial cells is initiated with binding of the proangiogenic factors such as VEGF, FGF, PDGF to their receptors followed by transduction of angiogenic signaling in the cells [60]. VEGF is regarded as one of the earliest and important signals to stimulate multistep cascade of tumor angiogenesis by promoting endothelial cell proliferation and migration [61]. Luteolin significantly (P<0.05) inhibited the elevated level of VEGF in PC-3 cells. VEGFR2 is essential for the morphogenesis of vascular endothelium and is the primary receptor mediating the angiogenic activity of VEGF through distinct signal transduction pathways that regulate endothelial cell proliferation, migration, differentiation and tube formation [62]–[64]. The VEGFR2 signaling pathway is a promising target of angiogenesis, because it is a common pathway for tumor-induced angiogenesis [65]. VEGFR2 mediates the majority of the downstream effects of VEGF in angiogenesis. Activation of VEGFR2 induced by VEGF leads to the stimulation of various intracellular signaling cascades, including activation of AKT, ERK, mTOR, and p70S6K pathways [66], [67]. Interruption of VEGFR2 signaling is thought to be necessary for tumor angiogenesis and macroscopic solid tumor growth [68]. In the present study, we have demonstrated that luteolin is able to dramatically inhibit VEGF-induced VEGFR2 activation.

AKT is a serine/threonine kinase that plays a central role in a range of cellular functions including cell growth, proliferation, migration, protein synthesis, transcription procedure and survival and angiogenesis [69], [70]. AKT regulates endothelial nitric oxide (NO) synthase (eNOS) activation [71], which in turn stimulates vasodilation, vascular remodeling and angiogenesis [70]. AKT signaling stimulates the production of hypoxia inducible factor α (HIFα) transcription factors, which mediates secretion of VEGF and other growth factors which are important proangiogenic factors [72], [73]. The mammalian target of rapamycin (mTOR) is a protein kinase of the PI3K/Akt signalling pathway with a central role in the control of cell proliferation, survival, mobility and angiogenesis. Dysregulation of mTOR pathway has been found in many human tumors; therefore, the mTOR pathway is considered an important target for the development of new anticancer drugs [74]. One of the functions of Akt is phosphorylation and activation of mTOR. Subsequently, activated mTOR regulates p70S6K phosphorylation and activation [75].

The extracellular signal-related kinase 1/2 (ERK1/2), one of the major targets of the mitogen-activated protein kinase (MAPK) signaling pathway, has been implicated in the regulation of angiogenesis for various functions including cell proliferation, migration, and survival [35]. Upon the extracellular growth factor stimulation, the activated ERK regulates its many substrates, such as NF-κB and c-Jun, thereby regulating angiogenesis. ERK is also necessary for eNOS activation [76]. P70S6K kinase (p70S6K) and S6 ribosomal protein (S6RP) are proteins downstream of AKT, and activation of p70S6K and S6RP stimulates protein synthesis and promotes cell growth and proliferation [77]. The present study luteolin inhibited VEGF-dependent phosphorylation of AKT, ERK, mTOR, and P70S6K in a dose dependent manner. In conclusion, the present study shows that luteolin is a potent inhibitor of angiogenesis *in vitro, ex vivo* and *in vivo*. Luteolin treatment inhibited the activation of VEGF-R2 and thereby suppressed the AKT/ERK/mTOR/P70S6K mediated angiogenesis signaling pathways.
